# The Role of Native T1 Mapping in the Diagnosis of Myocarditis in a Real-World Setting

**DOI:** 10.3390/jcm9123810

**Published:** 2020-11-25

**Authors:** Johannes Kersten, Tobias Heck, Laura Tuchek, Wolfgang Rottbauer, Dominik Buckert

**Affiliations:** Department of Internal Medicine II, University of Ulm, 89081 Ulm, Germany; tobias.heck@uni-ulm.de (T.H.); laura.tuchek@uni-ulm.de (L.T.); wolfgang.rottbauer@uniklinik-ulm.de (W.R.); dominik.buckert@uni-ulm.de (D.B.)

**Keywords:** cardiovascular magnetic resonance (CMR), T1 mapping, acute myocarditis, diagnostic performance

## Abstract

Background: This prospective single-center study sought to investigate the impact of cardiovascular magnetic resonance (CMR) on the diagnosis of myocarditis, with special attention given to absolute T1 values and defined cutoff values. Methods: All patients referred to our center with the suspicion of an inflammatory myocardial disease were diagnosed by a consensus expert consortium blinded to CMR findings. Classical Lake Louise criteria were then used to confirm or change the diagnosis. Results: Of a total of 149 patients, 15 were diagnosed with acute myocarditis without taking CMR findings into account. Acute myocarditis was excluded in 91 patients, whereas 42 cases were unclear. Using classical Lake Louise criteria, an additional 35 clear diagnoses were made, either confirming or excluding myocarditis. In the remaining patients, there was no further increase in definitive diagnoses using T1 measurements. The diagnostic performance of T1 mapping in distinguishing acute myocarditis patients from healthy controls was good (area under the curve (AUC) 0.835, cutoff value 1019 ms, sensitivity 73.7%, specificity 72.4%). In the group of patients with suspected and then excluded myocarditis, the cutoff value had a false-positive rate of 56.6%. Conclusions: Acute myocarditis should be diagnosed on the basis of clinical and imaging factors, whereas T1 mapping could be helpful, especially for excluding acute myocarditis.

## 1. Introduction

Myocarditis is a challenging disease due to its broad clinical manifestations and its demanding diagnostic workup with frequent differential diagnoses [1,2]. Cardiovascular magnetic resonance (CMR) imaging has developed into a cornerstone of the workup of patients with suspected inflammatory myocardial diseases [2,3,4,5]. This is due to the fact that CMR offers the possibility of imaging the major processes of inflammation: hyperemia, edema, and necrosis/fibrosis. They are illustrated by early gadolinium enhancement, T2-weighted images, and late gadolinium enhancement (LGE)—the so-called classical Lake Louise criteria. Today, these techniques have been replaced by parametric mapping of native T1 and T2 and the measurement of extracellular volume (ECV). These mapping techniques allow a good inter- and intra-individual comparability regardless of, for example, systemic inflammatory processes. However, although standardized mapping techniques have improved diagnostic accuracy [3,6,7,8,9,10], CMR-based tissue characterization is still open to debate, as a recent meta-analysis found no superiority to classical Lake Louise criteria [11].

Myocardial T1 mapping measures the longitudinal relaxation time as a tissue-specific value. It mainly depends on the composition of protons in water (increases T1) or in complex hydrocarbons as fat (decreases T1). In contrast to LGE (as part of the classical Lake Louise criteria), diffuse changes in tissue composition can also be recognized. The values are influenced by the field strength, vendors, and acquisition technique [6,9,12]. For this reason, there are no reliable cutoff values for the diagnosis of myocarditis. Each center must define its own standard and cutoff values. Moreover, there is a gray area in every diagnostic test. Mildly ill people with moderate symptoms and almost normal laboratory parameters, who particularly need sensible rule-in/rule-out diagnostics, usually end up in this gray area. Another question is whether the results of parametric mapping indeed help to differentiate myocarditis from other diseases that may also be closely related to changes in mapping. The aims of this study, therefore, were to determine cutoff values for T1 mapping in the diagnosis of myocarditis with specialized sequences, and to evaluate their impact on clinical decision making in the daily routine.

## 2. Materials and Methods

### 2.1. Study Population and Study Design

All patients undergoing CMR for a potential inflammatory myocardial disease were enrolled in this prospective, single-center study. This included patients with the suspicion of myocarditis, considerations of a differential inflammatory disease diagnosis (e.g., sarcoidosis), follow-up examinations after recent myocarditis—in other words, cases involving CMR examination where acute myocarditis had to be ruled out. Patients were enrolled from the daily routine or upon referral to our center. Enrollment took place from December 2016 to October 2018. All included patients provided written informed consent. There were no exclusion criteria aside from contraindications for CMR or contrast agents (e.g., claustrophobia, pregnancy, or severe impairment of kidney function). For inclusion, no coronary angiograms or endomyocardial biopsies (EMB) were obligatory. These invasive diagnostics were carried out only when necessary according to current guidelines.

A control group of healthy volunteers underwent the same CMR protocol as the patients, except for the LGE sequence to calculate cutoff values for T1 mapping. The inclusion criteria were no history of cardiac symptoms or previous cardiac, malignant, or rheumatic diseases according to a standardized questionnaire, normal cardiac function in cine imaging, normal troponin T and NT-proBNP values, and normal findings in a 12-lead electrocardiogram (ECG).

**Figure 1:**
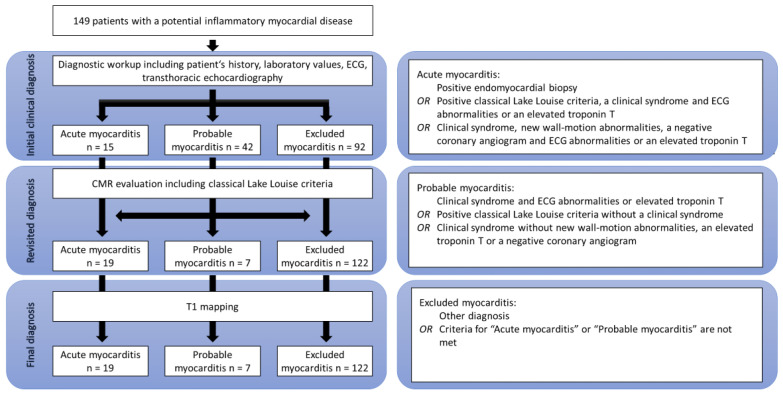
Patient enrollment, diagnostic workup, and diagnostic criteria.

In the patient cohort, the diagnosis of acute myocarditis was made by a consortium expert in consensus, using all available patient management data, including patient history, laboratory data such as troponin T and NT-proBNP levels, ECG abnormalities such as branch blocks or repolarization disorders, and echocardiographic findings such as wall motion abnormalities. As some patients were only referred to our CMR unit and treated elsewhere, not all information was available for every patient. In such cases, missing data were excluded from consideration. Based on adopted criteria proposed by Bonaca and colleagues, the patients were assigned to three possible diagnosis groups: (1) acute myocarditis, (2) probable acute myocarditis, and (3) acute myocarditis excluded (see Figure 1) [13].

In the first step, the expert committee was blinded to CMR findings, including mapping values. In the next step, to evaluate the impact of CMR and classical Lake Louise criteria on diagnostic decision making, CMR findings were used by the same committee, which was still blinded to mapping values, to establish a final diagnosis and assign the patients to the three groups. This diagnosis formed the basis for the calculation of cutoff values since it is the standard of care in daily routine. The study plan is seen in Figure 1.

In the last step, the generated cutoff values were used in the entire patient cohort (including non-myocarditis patients) to evaluate their specificity in a real-world setting.

The study was approved by the local ethics committee (approval number 238/16).

### 2.2. CMR

CMR was performed on a 1.5 T scanner (Achieva, Philips, Best, Netherlands). The CMR protocol consisted of balanced steady state free precession (bSSFP) cine images in short- and long-axis orientations (repetition time 3.4 ms, echo time 1.7 ms, slice thickness 8 mm, no interslice gap, acquisition in end-expiration breath-hold). Additionally, T2-weighted images using a turbo spin-echo (TSE) sequence and T1 mapping using a specially validated modified Look-Locker inversion (MOLLI) recovery sequence in the 5(3)3 scheme were acquired. With the exception of the control group of healthy volunteers, after a Look-Locker sequence for individual adjustment of the inversion time, late gadolinium enhancement images (repetition time 7.1 ms, echo time 3.2 ms, slice thickness 8 mm, respiratory navigator) were obtained 10 min after administering gadoterate meglumine (Dotarem^®^; Guerbet, Villepinte, France) in a dose of 0.2 mmol/kg of body weight.

All CMR images were analyzed by two reviewers in consensus, using commercially available software (cvi42; Circle Cardiovascular Imaging Inc., Calgary, AB, Canada). The evaluation was performed before the clinical diagnosis by the expert committee.

### 2.3. Statistical Analysis

Statistical analysis was performed using IBM SPSS Statistics 25 (IBM, Armonk, NY, USA). Continuous variables were expressed as means ± standard deviations, and categorical values were expressed as numbers and percentages. After assessing the normality of data distribution using the Shapiro–Wilk test, the Student’s *t*-test was used for normally distributed variables. A two-tailed *p*-value of <0.05 was considered statistically significant. Diagnostic accuracy was evaluated by areas under the curve (AUC) from receiver operating characteristic (ROC) analyses. For this purpose, the final diagnosis was used according to the diagnostic criteria in Figure 1 after evaluation for all available clinical, laboratory, and imaging data. Diagnostic sensitivity and specificity were calculated accordingly.

## 3. Results

### 3.1. Study Population and Diagnosis

A total of 149 patients (55 females and 94 males) were included in the study. Their mean age was 45.7 ± 17.3 years. A coronary angiogram was performed in 41 patients (27.5%) with one diagnosis of a severe coronary artery disease. In addition, in nine patients, an EMB was done with the histopathological proof of three cases of myocarditis. In the first step, 15 patients were definitively diagnosed and 42 were tentatively diagnosed with acute myocarditis. Acute myocarditis was excluded in 92 patients. In the next step, using classical Lake Louise criteria with T2-weighted images and LGE, a total of 19 patients were definitively diagnosed with acute myocarditis. In 122 patients, myocarditis was excluded through differential diagnoses. This included patients with a final diagnosis of diseases such as Takotsubo cardiomyopathy, dilative cardiomyopathy, and cardiac amyloidosis, as well as apparently healthy individuals. After this step, only seven patients remained in the probable acute myocarditis diagnosis group, which represents an 83.3% relative reduction. However, no diagnosis was changed from definite to excluded acute myocarditis and vice versa based on CMR imaging alone (Figure 1). In fact, as seen in Table 1, a non-infarct-like LGE was apparent in all patients with a final myocarditis diagnosis.

### 3.2. T1 Measurements

T1 values were highest in the definite and probable acute myocarditis groups, with no statistically significant difference between them (1064.3 ± 58.4 vs. 1064.6 ± 71.2; *p* = 0.992). Statistically significant differences were observed between the confirmed acute myocarditis and excluded acute myocarditis (1025.6 ± 70.8; *p* = 0.015) and control groups (1001.8 ± 27.1; *p* < 0.001). An evaluation for volumetry, classical Lake Louise criteria, and T1 values is seen in Table 1 (the *p*-values refer to comparisons with the definite acute myocarditis group).

**Figure 2:**
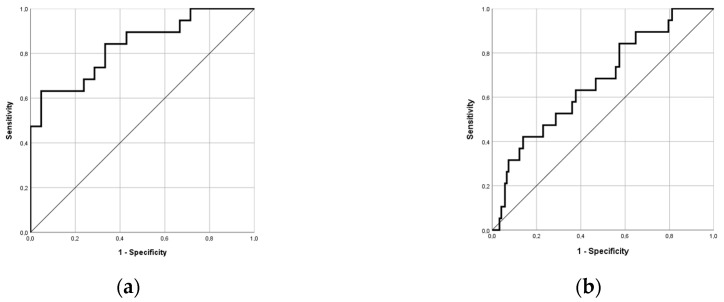
Receiver operating characteristic analysis of the accuracy of (**a**) T1 mapping in the diagnosis of acute myocarditis with an area under the curve of 0.835 and (**b**) T1 mapping in differentiating acute myocarditis from non-myocarditis patients with an area under the curve of 0.670.

ROC analysis showed that T1 measurements had good diagnostic performance in differentiating between acute myocarditis patients and healthy individuals, with an AUC of 0.835 (95% confidence interval: 0.71, 0.96). The accordingly calculated cutoff value was 1019 ms, with 73.7% sensitivity and 72.4% specificity (Figure 2a). However, in the entire cohort of patients with and without myocarditis in a real-world setting, the cutoff value showed a false-positive rate of 56.6% (43.4% specificity). Another ROC analysis focusing on the differentiation between acute myocarditis patients and excluded myocarditis patients produced similar results, with an AUC of only 0.670 (Figure 2b). The calculated cutoff value of 983 ms showed a negative predictive value of 95.8%.

**Figure 3:**
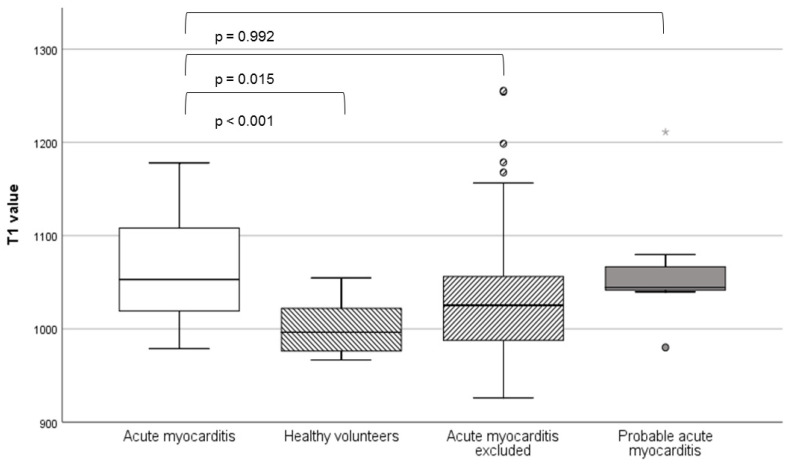
T1 values of patients with confirmed, probable, and excluded acute myocarditis and healthy controls. The box plot shows the median and the upper and lower quartiles; the whiskers show the minimum and maximum.

Regarding the range of T1 values, we found a very broad scattering rate with several outliers in the excluded acute myocarditis group (Figure 3).

In the group of patients who were tentatively diagnosed with possible acute myocarditis in the first step, the T1 value was higher in patients who were eventually definitively diagnosed with acute myocarditis than in those in whom myocarditis was eventually excluded. However, the difference was not statistically significant (1090.5 ± 72.9 vs. 1021.9 ± 81.5; *p* = 0.239), most likely due to the small sample size.

## 4. Discussion

In our study, the gray area of non-definitive diagnosis was narrowed substantially using classical Lake Louise criteria. Furthermore, T1 mapping and the calculated cutoff value showed good performance in differentiating acute myocarditis patients from healthy controls but low diagnostic value in the entire cohort, which is representative of daily routine. This can be attributed to the broad range of other conditions related to high T1 values.

The improved diagnostic selectivity with CMR reflects its incremental role in the diagnosis of acute myocarditis emphasized in the literature [1,2,4,14,15]. LGE is the main driver of the diagnostic performance of the classical Lake Louise criteria [11]. However, in our study, no diagnoses were completely changed after CMR assessment. Only unclear cases were definitively diagnosed as acute myocarditis or non-myocarditis, at a rate of 23.7%.

T1 mapping did not further increase the frequency of myocarditis diagnosis in our study. This might have been a result of the very small number of unclear diagnoses. Conversely, the synopsis of patient history, laboratory data such as troponin T and NT-proBNP, ECG abnormalities, coronary angiography, echocardiography, and CMR seems to offer a level of accuracy that is hard to surpass.

Several studies have shown that T1 has high diagnostic accuracy [3,9,10,16,17,18,19]. Using a shortened MOLLI sequence and a vendor different from that in our study, Ferreira et al. reported a T1 value of 990 ms with an AUC of 0.95 [16]. Unlike our study, only patients with clinically unquestionable myocarditis were included in that study. Other studies have likewise evaluated T1 diagnostic accuracy only in patients with a clinical diagnosis of severe myocarditis [7,10,18,19]. A meta-analysis by Pan et al. found that native T1 has superior sensitivity and comparable specificity to the classical Lake Louise criteria [11]. Our study suggests that despite all the positive data, the sensitivity and specificity of T1 mapping in a heterogeneous clinical cohort is still questionable. We found a good negative predictive value of 95.8% at a T1 time of 983 ms, which is as high as that of the d-dimer test for deep vein thrombosis [20]. This suggests that native T1 may play a role in exclusion rather than diagnosis. Its independence from a contrast agent offers a considerable advantage. LGE, the main driver of the classical Lake Louise criteria, depends on a gadolinium-based contrast agent and could possibly be replaced by native T1 mapping in patients for whom gadolinium is contraindicated, such as pregnant or breastfeeding women or patients with a severe kidney injury.

A combination of T1 and T2 measurements may have a greater impact on clinical decision making. T2 mapping reflects myocardial edema, seen in acute inflammatory myocardial diseases. In our cohort, due to the late implementation of a robust gradient and spin–echo sequence (GraSE) during patient enrollment, there were T2 values only for 4 patients with confirmed and 31 patients with excluded myocarditis. Even in this small group, a clear tendency toward different T2 values (67.5 ± 7.1 vs. 54.3 ± 4.1; *p* = 0.055) could be seen. Other studies also show a good correlation between T2 values, edema, and disease activity in inflammatory diseases [21,22,23]. In chronic myocarditis, the role of T2 mapping is unclear [8,9,13].

A limitation of our study was the lack of post-contrast T1 mapping and the calculation of ECV accordingly. ECV could provide adjunctive information for other forms of tissue characterization [10,11]. The diagnostic accuracy under the circumstances of daily practice needs further investigation.

In the group of patients with finally excluded myocarditis, there was a high percentage of pericardial effusions and wall motion abnormalities. In a detailed revision, a small and clinically irrelevant pericardial effusion (mostly as an incidental finding), or a reduced left ventricular function at a global or a regional level had led to the primary assumption of a myocarditis in most of these patients. Thus, the higher numbers were a result of selection bias.

We did not use EMB as standard in our study, even though it is the diagnostic gold standard for myocarditis. In our opinion, the absence of a diagnostic EMB better reflects the daily clinical routine, where acute myocarditis is not diagnosed by this investigation alone. EMB is highly susceptible to a sampling error, which may result in a high false-negative rate [24,25,26]. It is therefore only definitely recommended in cases of new-onset heart failure (less than two weeks) and immanent clinical symptoms, such as hemodynamic instability or ventricular arrhythmias [27]. In these rarer cases, an EMB is potentially therapy-changing (e.g., by proving giant cell myocarditis) [28]. Because of these diagnostic gaps in EMB, we recommend using other diagnostic concepts for clinical and scientific purposes, such as the criteria proposed by Bonaca et al. [13]. In consideration of the current guidelines, EMBs were performed only in a few cases in our cohort.

## 5. Conclusions

CMR is a valuable tool in the diagnostic workup for acute myocarditis. Despite its diagnostic performance using idealized normal values obtained from healthy individuals, T1 measurements could not improve diagnosis in our cohort. A synopsis of different CMR parameters and clinical and laboratory data can most likely lead to an adequate diagnosis of acute myocarditis. In the end, diagnosis should be established by the clinical cardiologist and not the imaging specialist.

A critical discussion on promising methods such as T1 mapping is also required. We therefore recommend larger studies to reveal the actual diagnostic impact of T1 mapping on clinical decision making. The classical Lake Louise criteria are still a useful tool even when parametric mapping is performed.

## Figures and Tables

**Table 1:** Patient demographics, volumetry, classical Lake Louise criteria, and T1 measurements.

Variables	Definite Acute Myocarditis (*n* = 19)	Probable Acute Myocarditis (*n* = 7)	Acute Myocarditis Excluded (*n* = 122)	Healthy Controls (*n* = 21)
Demographics				
Age, years	35.1 ± 18.9	47.9 ± 15.3 (*p* = 0.121)	47.2 ± 16.7 (*p* = 0.004)	41.3 ± 14.0 (*p* = 0.240)
Females, *n* (%)	8 (42.1)	3 (42.9)	44 (36.1)	11 (52.4)
Body mass index, kg/cm^2^	25.6 ± 5.4	27.3 ± 6.5 (*p* = 0.522)	25.8 ± 4.1 (*p* = 0.902)	26.9 ± 4.6 (*p* = 0.435)
Volumetry and function				
LVEF, %	60.7 ± 6.6	54.9 ± 9.8 (*p* = 0.208)	57.8 ± 11.4 (*p* = 0.135)	65.3 ± 6.7 (*p* = 0.044)
LVEDV, mL	143.7 ± 30.4	158.4 ± 34.4 (*p* = 0.375)	160.4 ± 56.1 (*p* = 0.067)	155.7 ± 32.1 (*p* = 0.222)
LVEDVI, mL/m^2^	75.0 ± 11.4	80.9 ± 15.5 (*p* = 0.419)	82.4 ± 25.1 (*p* = 0.076)	78.7 ± 15.9 (*p* = 0.337)
Stroke volume, mL	86.5 ± 16.2	86.0 ± 19.3 (*p* = 0.953)	88.5 ± 24.4 (*p* = 0.652)	100.3 ± 16.7 (*p* = 0.173)
LV mass, g	97.1 ± 32.1	113.9 ± 31.6 (*p* = 0.286)	101.1 ± 41.0 (*p* = 0.634)	112.8 ± 35.5 (*p* = 0.174)
RVEF, %	58.3 ± 7.4	59.7 ± 6.9 (*p* = 0.680)	59.3 ± 9.7 (*p* = 0.619)	59.9 ± 6.8 (*p* = 0.512)
Classical Lake Louise criteria				
T2-weighted imaging, *n* (%)	5 (26.3)	1 (14.3)	3 (2.4)	-
Early gadolinium enhancement, *n* (%)	8 (42.1)	3 (42.9)	17 (13.9)	-
Late gadolinium enhancement, *n* (%)	19 (100)	2 (28.6)	4 (3.3)	-
Pericardial effusion, *n* (%)	6 (31.6)	0 (0)	15 (12.3)	-
Systolic LV wall motion abnormality, *n* (%)	3 (15.8)	4 (57.1)	31 (25.4)	-
T1 mapping, ms	1064.3 ± 58.4	1064.6 ± 71.2 (*p* = 0.992)	1025.6 ± 70.8 (*p* = 0.015)	1001.8 ± 27.1 (*p* < 0.001)

The *p*-values refer to comparisons with the definite acute myocarditis group. Continuous variables are expressed as means ± standard deviations. Categorical variables are expressed as numbers (percentages).

## References

[1] Fung G., Luo H., Qiu Y., Yang D., McManus B. (2016). Myocarditis. Circ. Res..

[2] Caforio A.L.P., Pankuweit S., Arbustini E., Basso C., Gimeno-Blanes J., Felix S.B., Fu M., Heliö T., Heymans S., Jahns R. (2013). Current state of knowledge on aetiology, diagnosis, management, and therapy of myocarditis: A position statement of the European Society of Cardiology Working Group on Myocardial and Pericardial Diseases. Eur. Heart J..

[3] Ferreira V.M., Schulz-Menger J., Holmvang G., Kramer C.M., Carbone I., Sechtem U., Kindermann I., Gutberlet M., Cooper L.T., Liu P. (2018). Cardiovascular Magnetic Resonance in Nonischemic Myocardial Inflammation: Expert Recommendations. J. Am. Coll. Cardiol..

[4] Friedrich M.G., Sechtem U., Schulz-Menger J., Holmvang G., Alakija P., Cooper L.T., White J.A., Abdel-Aty H., Gutberlet M., Prasad S. (2009). Cardiovascular magnetic resonance in myocarditis: A JACC White Paper. J. Am. Coll. Cardiol..

[5] Kersten J., Güleroglu A.M., Rosenbohm A., Buckert D., Ludolph A.C., Hackenbroch C., Beer M., Bernhardt P. (2020). Myocardial involvement and deformation abnormalities in idiopathic inflammatory myopathy assessed by CMR feature tracking. Int. J. Cardiovasc. Imaging.

[6] Haaf P., Garg P., Messroghli D.R., Broadbent D.A., Greenwood J.P., Plein S. (2016). Cardiac T1 Mapping and Extracellular Volume (ECV) in clinical practice: A comprehensive review. J. Cardiovasc. Magn. Reson..

[7] Luetkens J.A., Homsi R., Sprinkart A.M., Doerner J., Dabir D., Kuetting D.L., Block W., Andrié R., Stehning C., Fimmers R. (2016). Incremental value of quantitative CMR including parametric mapping for the diagnosis of acute myocarditis. Eur. Heart J. Cardiovasc. Imaging.

[8] Lurz P., Luecke C., Eitel I., Föhrenbach F., Frank C., Grothoff M., de Waha S., Rommel K.-P., Lurz J.A., Klingel K. (2016). Comprehensive Cardiac Magnetic Resonance Imaging in Patients with Suspected Myocarditis: The MyoRacer-Trial. J. Am. Coll. Cardiol..

[9] Baessler B., Luecke C., Lurz J., Klingel K., Das A., von Roeder M., de Waha-Thiele S., Besler C., Rommel K.-P., Maintz D. (2019). Cardiac MRI and Texture Analysis of Myocardial T1 and T2 Maps in Myocarditis with Acute versus Chronic Symptoms of Heart Failure. Radiology.

[10] Radunski U.K., Lund G.K., Stehning C., Schnackenburg B., Bohnen S., Adam G., Blankenberg S., Muellerleile K. (2014). CMR in patients with severe myocarditis: Diagnostic value of quantitative tissue markers including extracellular volume imaging. JACC Cardiovasc. Imaging.

[11] Pan J.A., Lee Y.J., Salerno M. (2018). Diagnostic Performance of Extracellular Volume, Native T1, and T2 Mapping Versus Lake Louise Criteria by Cardiac Magnetic Resonance for Detection of Acute Myocarditis: A Meta-Analysis. Circ. Cardiovasc. Imaging.

[12] Dabir D., Child N., Kalra A., Rogers T., Gebker R., Jabbour A., Plein S., Yu C.-Y., Otton J., Kidambi A. (2014). Reference values for healthy human myocardium using a T1 mapping methodology: Results from the International T1 Multicenter cardiovascular magnetic resonance study. J. Cardiovasc. Magn. Reson..

[13] Bonaca M.P., Olenchock B.A., Salem J.-E., Wiviott S.D., Ederhy S., Cohen A., Stewart G.C., Choueiri T.K., Di Carli M., Allenbach Y. (2019). Myocarditis in the Setting of Cancer Therapeutics: Proposed Case Definitions for Emerging Clinical Syndromes in Cardio-Oncology. Circulation.

[14] Hundley W.G., Bluemke D.A., Finn J.P., Flamm S.D., Fogel M.A., Friedrich M.G., Ho V.B., Jerosch-Herold M., Kramer C.M., Manning W.J. (2010). ACCF/ACR/AHA/NASCI/SCMR 2010 expert consensus document on cardiovascular magnetic resonance: A report of the American College of Cardiology Foundation Task Force on Expert Consensus Documents. J. Am. Coll. Cardiol..

[15] Kotanidis C.P., Bazmpani M.-A., Haidich A.-B., Karvounis C., Antoniades C., Karamitsos T.D. (2018). Diagnostic Accuracy of Cardiovascular Magnetic Resonance in Acute Myocarditis: A Systematic Review and Meta-Analysis. JACC Cardiovasc. Imaging.

[16] Ferreira V.M., Piechnik S.K., Dall’Armellina E., Karamitsos T.D., Francis J.M., Ntusi N., Holloway C., Choudhury R.P., Kardos A., Robson M.D. (2013). T(1) mapping for the diagnosis of acute myocarditis using CMR: Comparison to T2-weighted and late gadolinium enhanced imaging. JACC Cardiovasc. Imaging.

[17] Moon J.C., Messroghli D.R., Kellman P., Piechnik S.K., Robson M.D., Ugander M., Gatehouse P.D., Arai A.E., Friedrich M.G., Neubauer S. (2013). Myocardial T1 mapping and extracellular volume quantification: A Society for Cardiovascular Magnetic Resonance (SCMR) and CMR Working Group of the European Society of Cardiology consensus statement. J. Cardiovasc. Magn. Reson..

[18] Dabir D., Vollbrecht T.M., Luetkens J.A., Kuetting D.L.R., Isaak A., Feisst A., Fimmers R., Sprinkart A.M., Schild H.H., Thomas D. (2019). Multiparametric cardiovascular magnetic resonance imaging in acute myocarditis: A comparison of different measurement approaches. J. Cardiovasc. Magn. Reson..

[19] Hinojar R., Foote L., Arroyo Ucar E., Jackson T., Jabbour A., Yu C.-Y., McCrohon J., Higgins D.M., Carr-White G., Mayr M. (2015). Native T1 in discrimination of acute and convalescent stages in patients with clinical diagnosis of myocarditis: A proposed diagnostic algorithm using CMR. JACC Cardiovasc. Imaging.

[20] Philbrick J.T., Heim S. (2003). The d-dimer test for deep venous thrombosis: Gold standards and bias in negative predictive value. Clin. Chem..

[21] Thavendiranathan P., Walls M., Giri S., Verhaert D., Rajagopalan S., Moore S., Simonetti O.P., Raman S.V. (2012). Improved detection of myocardial involvement in acute inflammatory cardiomyopathies using T2 mapping. Circ. Cardiovasc. Imaging.

[22] Hinojar R., Foote L., Sangle S., Marber M., Mayr M., Carr-White G., D’Cruz D., Nagel E., Puntmann V.O. (2016). Native T1 and T2 mapping by CMR in lupus myocarditis: Disease recognition and response to treatment. Int. J. Cardiol..

[23] Baeßler B., Schaarschmidt F., Treutlein M., Stehning C., Schnackenburg B., Michels G., Maintz D., Bunck A.C. (2017). Re-evaluation of a novel approach for quantitative myocardial oedema detection by analysing tissue inhomogeneity in acute myocarditis using T2-mapping. Eur. Radiol..

[24] Korkusuz H., Esters P., Huebner F., Bug R., Ackermann H., Vogl T.J. (2010). Accuracy of cardiovascular magnetic resonance in myocarditis: Comparison of MR and histological findings in an animal model. J. Cardiovasc. Magn. Reson..

[25] Lie J.T. (1988). Myocarditis and endomyocardial biopsy in unexplained heart failure: A diagnosis in search of a disease. Ann. Intern. Med..

[26] Borchert B., Lawrenz T., Bartelsmeier M., Röthemeyer S., Kuhn H., Stellbrink C. (2007). Utility of endomyocardial biopsy guided by delayed enhancement areas on magnetic resonance imaging in the diagnosis of cardiac sarcoidosis. Clin. Res. Cardiol..

[27] Cooper L.T., Baughman K.L., Feldman A.M., Frustaci A., Jessup M., Kuhl U., Levine G.N., Narula J., Starling R.C., Towbin J. (2007). The role of endomyocardial biopsy in the management of cardiovascular disease: A scientific statement from the American Heart Association, the American College of Cardiology, and the European Society of Cardiology Endorsed by the Heart Failure Society of America and the Heart Failure Association of the European Society of Cardiology. Eur. Heart J..

[28] Larsen B.T., Maleszewski J.J., Edwards W.D., Cooper L.T., Sobonya R.E., Thompson V.E., Duckett S.G., Peebles C.R., Simpson I.A., Tazelaar H.D. (2013). Atrial giant cell myocarditis: A distinctive clinicopathologic entity. Circulation.

